# Precision Nutrition: A Review of Personalized Nutritional Approaches for the Prevention and Management of Metabolic Syndrome

**DOI:** 10.3390/nu9080913

**Published:** 2017-08-22

**Authors:** Juan de Toro-Martín, Benoit J. Arsenault, Jean-Pierre Després, Marie-Claude Vohl

**Affiliations:** 1 Institute of Nutrition and Functional Foods (INAF), Laval University, Quebec City, QC G1V 0A6, Canada; juan.de-toro-martin.1@ulaval.ca; 2 School of Nutrition, Laval University, Quebec City, QC G1V 0A6, Canada; 3 Department of Medicine, Faculty of Medicine, Laval University, Quebec City, QC G1V 0A6, Canada; benoit.arsenault@criucpq.ulaval.ca; 4 Quebec Heart and Lung Institute, Quebec City, QC G1V 4G5, Canada; jean-pierre.despres@criucpq.ulaval.ca; 5 Department of Kinesiology, Faculty of Medicine, Laval University, Quebec City, QC G1V 0A6, Canada

**Keywords:** precision nutrition, nutrigenomics, physical activity, deep phenotyping, metabolomics, gut microbiota

## Abstract

The translation of the growing increase of findings emerging from basic nutritional science into meaningful and clinically relevant dietary advices represents nowadays one of the main challenges of clinical nutrition. From nutrigenomics to deep phenotyping, many factors need to be taken into account in designing personalized and unbiased nutritional solutions for individuals or population sub-groups. Likewise, a concerted effort among basic, clinical scientists and health professionals will be needed to establish a comprehensive framework allowing the implementation of these new findings at the population level. In a world characterized by an overwhelming increase in the prevalence of obesity and associated metabolic disturbances, such as type 2 diabetes and cardiovascular diseases, tailored nutrition prescription represents a promising approach for both the prevention and management of metabolic syndrome. This review aims to discuss recent works in the field of precision nutrition analyzing most relevant aspects affecting an individual response to lifestyle/nutritional interventions. Latest advances in the analysis and monitoring of dietary habits, food behaviors, physical activity/exercise and deep phenotyping will be discussed, as well as the relevance of novel applications of nutrigenomics, metabolomics and microbiota profiling. Recent findings in the development of precision nutrition are highlighted. Finally, results from published studies providing examples of new avenues to successfully implement innovative precision nutrition approaches will be reviewed.

## 1. Precision Nutrition 

### The Road to Tailored Dietary Advices

One of the ultimate goals of the promising field of precision nutrition is the design of tailored nutritional recommendations to treat or prevent metabolic disorders [1]. More specifically, precision nutrition pursuits to develop more comprehensive and dynamic nutritional recommendations based on shifting, interacting parameters in a person’s internal and external environment throughout life. To that end, precision nutrition approaches include, in addition to genetics, other factors such as dietary habits, food behavior, physical activity, the microbiota and the metabolome. Following the completion of the mapping of the Human Genome, a cumulative number of association studies have been performed in order to identify the genetic factors that may explain the inter-individual variability of the metabolic response to specific diets. In this sense, while numerous genes and polymorphisms have been already identified as relevant factors in this heterogeneous response to nutrient intake [2,3,4,5,6,7], clinical evidence supporting these statistical relationships is currently too weak to establish a comprehensive framework for personalized nutritional interventions in most cases [8]. Thus, although most of findings on this topic are still relatively far from giving their fully expected potential in terms of translation and application of this knowledge to precision nutrition [9], some of them have been successfully developed in both the public and the private sectors. On one hand, the hypolactasia diagnosis [10], the celiac disease ruling out [11] or the phenylketonuria screening [12], have allowed the implementation of tailored nutritional advices based on genetic makeup for years, i.e., avoiding lactose-, gluten- and phenylalanine-containing products to at-risk individuals. On the private sector, many companies are already offering genetic tests to customize diets based on the individual response to specific nutrients. For instance, that is the case of genetic tests based on the specific metabolism of caffeine (slow or fast metabolizers) [13,14], the predisposition to weight gain by saturated fat intake [15,16], or the increased risk of developing hypertension by high salt intake [17,18], among others. Together, these nutritional recommendations solely based on genetic background represents a straightforward approach to the concept of personalized nutrition. Although quite similar to the concept of precision nutrition, and sometimes interchangeable, the latter makes reference to a conceptual framework covering a wider set of individual features allowing an effective and dynamic nutritional approach [1]. Thus, while personalized nutrition based on genes is already being implemented successfully based on numerous research studies, such as the ones above mentioned, precision nutrition may still lack sufficient evidence for full implementation given its complexity, as will be reviewed below.

Regarding obesity and metabolic syndrome, recent published studies focusing on gene-environment interactions have revealed important insights about the impact of macronutrient intake in the association of genetic markers with metabolic health, fat mass accumulation or body composition. This is broadly relevant in precision nutrition, since results from these studies, focused on macronutrient intake, open the door to tailor efficiently diets based on the individual genetic makeup. In this regard, recent work by Goni et al. [19] analyzed the usefulness of a genetic risk score (GRS) on obesity prediction, and more interestingly, the impact of macronutrient intake in the predictive value of this GRS. The GRS was built as an additive summary measure of a set of 16 genetic variants (according to the number of risk alleles for each variant) previously associated with obesity (rs9939609, *FTO*; rs17782313, *MC4R*; rs1801282, *PPARG*; rs1801133, *MTHFR* and rs894160, *PLIN1*) and lipid metabolism disturbances (rs1260326, *GCKR*; rs662799, *APOA5*; rs4939833, *LIPG*; rs1800588l, *LIPC*, rs328, *LPL*; rs12740374, *CELSR2*; rs429358 and rs7412, *APOE*; rs1799983, *NOS3*; rs1800777, *CETP* and rs1800206, *PPARA*). After the validation of the GRS, i.e., high risk group (subjects having more than 7 risk alleles) showing increased body mass index (0.93 kg/m^2^ greater BMI), body fat mass (1.69% greater BFM), waist circumference (1.94 cm larger WC) and waist-to-hip ratio (0.01 greater WHR), significant interactions between macronutrient intake and GRS prediction values were observed. For instance, higher intake of animal protein was significantly associated with higher BFM in individuals within the high-risk GRS group (P_interaction_ = 0.032), whereas higher vegetable protein consumption showed a protective effect among subjects in the low-risk group (P_interaction_ = 0.003), as these individuals were characterized by a lower percentage of BFM [19]. Similar trends were reported by Rukh et al., where total protein intake was found to modulate GRS association with obesity in women (P_interaction_ = 0.039) [20]. Other studies on gene-macronutrients interactions, in which a GRS developed on the basis of BMI-associated single nucleotide polymorphisms (SNPs) was used, have revealed that high intake of sugar-sweetened beverages [21,22,23], fried foods [24] or saturated fatty-acids [25] are also able to modulate the risk to develop obesity. Altogether, these results suggest that the accumulation of common polymorphisms at loci known to influence body weight may influence one’s predisposition to gain weight when exposed to certain types of diets. 

Over the past recent years, it has become increasingly evident that the assessment of dietary patterns provides a more reliable picture of real food intake compared to the assessment of macronutrients intake considered in isolation. In this regard, a recent work focused on the effect of the obesity-associated *MC4R* gene on metabolic syndrome has revealed a relevant gene-diet interaction with dietary patterns [26]. In this case-control study, participants with metabolic syndrome from the Tehran Lipid and Glucose Study [27] were randomly matched with controls by age and sex, leading a total of 815 pairs. Healthy and western dietary patterns were identified by factor analysis based on 25 food groups extracted from a 168-item semi-quantitative food frequency questionnaire (FFQ). The healthy dietary pattern was characterized by high intake of vegetables, legumes, low fat dairy, whole grains, liquid oils and fruits, while the western dietary pattern consisted of high intake of soft drinks, fast foods, sweets, solid oils, red meats, salty snacks, refined grains, high fat dairy, eggs and poultry. Results from this study revealed that carriers of the rare allele in the *MC4R* gene and having the highest score of the western dietary pattern had increased risk (odds ratio—OR) of developing metabolic syndrome (*OR* = 1.71 (1.04–2.41); P_trend_ = 0.007), as compared to those having lower scores [26]. Similar gene-dietary pattern interactions were revealed in another study linking GRS with WHR and BMI, and different diet scores, ranging from healthier (whole grains, fish, fruits, vegetables, nuts/seeds) to unhealthier (red/processed meats, sweets, sugar-sweetened beverages and fried potatoes) [28]. Results from this study, where more than 68,000 participants from 18 different cohorts were used, showed nominally significant associations between diet score and WHR-GRS, with stronger genetic effect in subjects with a higher diet score (β_interaction_ (SE_interaction_) = 4.77 × 10^−5^ (2.32 × 10^−5^); P_interaction_ = 0.04), i.e., consuming healthier diets [28]. 

**Figure 1:**
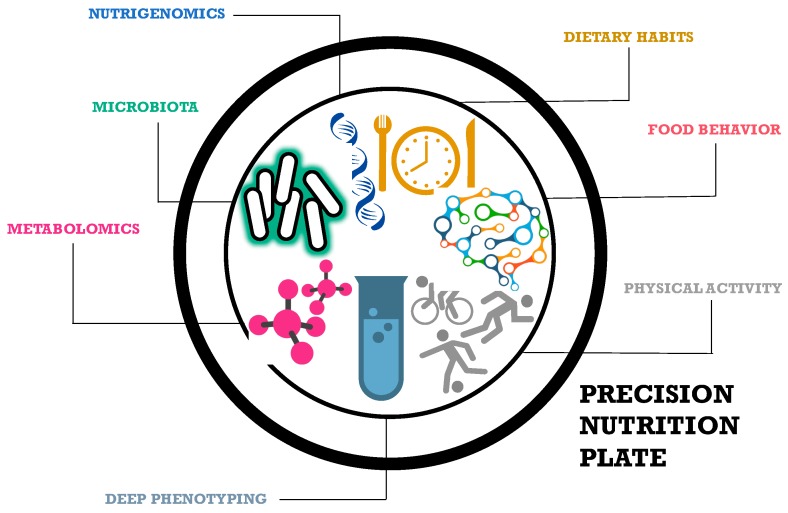
The *precision nutrition plate*. A schematic representation of the main factors worth to consider when approaching precision nutrition.

As above mentioned, the scientific community generally agrees that the future of precision nutrition will not be solely based on nutrigenetics [29]. Clearly, factors beyond genetics also need to be considered when designing personalized or tailored diets. In this regard, the usefulness of tailored dietary advices to adequately anticipate individual responses to nutritional intakes is one of the main goals of precision nutrition. In order to attain this goal, and as illustrated in the precision nutrition plate (Figure 1), determinants not only related to nutritional or genetic factors, e.g., lifestyle including physical activity (PA) habits, metabolomics or gut microbiomics, are also emerging as significant contributors that merit consideration in the field of precision nutrition [30,31,32].

This is the case of a recent study where the power of a machine-learning algorithm to predict postprandial glucose levels was tested [33]. In this study, the ability of an algorithm to forecast postprandial glycaemia as well as an expert-based prediction was reported. To do that, the high inter-individual variability of postprandial glycemic response was first revealed by using subcutaneous sensors that accurately monitored glucose levels (every 5 min during 7 full days) in a cohort of 800 subjects, resulting in over 1.5 million glucose measurements, corresponding to nearly 47,000 real-life meals and over 5000 standardized meals. A comprehensive profiling including data derived from a FFQ, sleep and PA habits, medical histories, anthropometric measures, blood tests and microbiota profiling was assessed for each participant. These features were then included in the prediction algorithm, which was first tested in the cohort of 800 subjects and further successfully validated in an independent cohort of 100 patients. Further analyses allowed the quantification of the partial contribution of each parameter of the algorithm, from meal nutrient content (carbohydrates, fat, dietary fibers, sodium) to microbiome-based features, in the prediction of postprandial glucose levels. Finally, the predictive performance of the algorithm was examined in a two-arm blinded randomized controlled trial with 26 new participants. In the first arm, after the 1-week profiling, 12 participants were sequentially assigned to an unhealthy or a healthy diet according to the postprandial glycemic responses predicted by the algorithm for each participant. In the second arm, 14 participants followed the same unhealthy and healthy diets, but dietary advices were given by a registered dietitian and a scientist experienced in analyzing continuous glucose monitoring data. The tailored dietary advice in both the predictor and the expert arms resulted in a significant decrease of postprandial glucose levels when participants were assigned to the healthy diet. More specifically, the correlation between postprandial glucose levels measured during the profiling and the intervention weeks was 0.7 in the expert arm, and it reached 0.8 with the algorithm-predicted values. These results, in spite of providing support for the potential of this personalized nutrition approach, should be taken with caution until further studies are completed, since some observations mainly concerning the inter-individual variability in glycemic responses have been recently pointed out [34]. In any case, such an innovative prediction algorithm, which utilizes clinical, nutritional and lifestyle variables, as well as microbiome profiles as input parameters, exemplifies the great possibilities offered by these sophisticated methods for the further implementation of precision nutrition.

**Figure 2:**
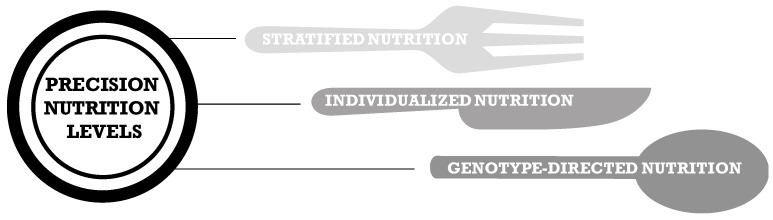
The three levels of precision nutrition according to the International Society of Nutrigenetics/Nutrigenomics (ISNN) [29].

According to the International Society of Nutrigenetics/Nutrigenomics (ISNN), the future of precision nutrition should be discussed at three levels: stratification of conventional nutritional guidelines into population subgroups by age, gender and other social determinants, individual approaches issued from a deep and refined phenotyping, and a genetic-directed nutrition based on rare genetic variants having high penetrance and impact on individuals’ response to particular foods [29] (Figure 2). This categorization of precision nutrition pillars includes a more in-depth exploration of the challenges that nutrition science must face in next years to evolve in the context of an increasing prevalence of obesity and associated metabolic disorders, resulting largely from the wide-scale adoption of unhealthy feeding behaviors in an *obesogenic* food environment in which it has become increasingly difficult to adhere to healthy dietary patterns.

In this regard, a better understanding of the inter-individual variability in the response to diet has been recently identified by the American Society of Nutrition (ASN) as one of the six top research priorities to be addressed in nutrition science to face the forthcoming challenges in population health management [35]. Moreover, the ASN has identified the required tools to attain these research needs for enabling an accurate nutritional impact prediction on health (omics technologies), an enhanced patient information survey (bioinformatics and database management), and a suitable assessment of disease progression and patient response to a nutritional treatment (biomarkers, metabolomics, etc.). 

This paper will review the recent advances in the field of precision nutrition, with special emphasis on the novel approaches of dietary habits assessment, food behavior evaluation, PA monitoring, as well as on the novel techniques applied to deep phenotyping, metabotyping and microbiota profiling.

## 2. Dietary Habits

### Fine-Tuning Adherence

The main goal sought with nutritional interventions is to assess potential associations between feeding behaviors and metabolic outcomes such as body composition, insulin sensitivity and markers of the lipoprotein-lipid profile. These potentially causal relationships should then enable to draw conclusions on the clinical relevance of specific nutritional recommendations for population subgroups. Unfortunately, one of the most common obstacles that nutritional science needs to tackle when exploring such associations is that conventional nutritional intervention studies often lack the power to detect subtle effects of diet on metabolic parameters, either because of the short duration of such studies or by the small number of participants involved [36]. The problem resulting from lack of statistical power may be amplified by several additional issues among which inter-individual variability and limited adherence evaluation stand out as potential determinants of modest study outcomes and underestimation of diet effects [37]. Regarding the impact of nutrition on genetic makeup and vice versa, the capacity to accurately monitor food and energy intake remains a major challenge in precision nutrition research.

A better characterization of dietary habits throughout an intervention study will ultimately increase one’s chance of generating clear findings. This implies, however, thorough data acquisition in terms of individual food consumption and other factors that could affect adherence evaluation of a particular intervention [38]. The limitations of subjective and memory-based dietary assessment methods (M-BM), such as FFQ, 24-h dietary recall (24H), dietary record (DR) and dietary history (DH) have been known for a long time [39] and continue to be questioned today [40], with under- or over-reporting not being accounted for in many studies, which may lead to biased results in nutritional intervention studies. Other than recall bias inherent in self-reported data, limitations shown by subjective dietary assessment methods comprise the high cost and time-consuming of DH and of multiple 24H and DR, which could also drive to unintentionally changes in participants’ diet due to repeated measurements [41]. Since a reliable dietary assessment is key for interpreting diet-induced metabolic outcomes, many approaches aimed at overcoming these issues from different perspectives have been proposed. 

Two examples of novel dietary adherence methods are the Mediterranean Diet Adherence Screener (MEDAS) [42,43] and the Mediterranean Lifestyle index (MEDLIFE) [44]. The MEDAS consists of a simple 14-point-instrument to overcome the classical time-consuming FFQ. This time saver questionnaire allows a more robust estimation of Mediterranean diet adherence that can be used in clinical practice. The final MEDAS score ranges from 0 (worst adherence) to 14 (best adherence), according to 9 items from a previously validated index [45], plus three questions on Mediterranean food consumption frequency (nuts per week, sugar-sweetened beverages per day, and tomato sauce with garlic, onion and olive oil per week), and two more questions about Spanish Mediterranean food intake habits (olive oil as the principal source of fat for cooking and preference of white meats—chicken, rabbit, turkey—over red meats—beef, pork, etc.). The MEDAS has been validated within the Prevención with Dieta Mediterránea (PREDIMED) study [46], a primary prevention nutrition-intervention trial that will be presented below. Using a holistic approach, the MEDLIFE index is the first to include PA and social interaction to the classical assessment of food consumption [44]. The MEDLIFE index has been validated with previous diet quality indices, the Alternate Healthy Eating Index (AHEI) [47], the alternate Mediterranean Diet Index (aMED) [48] and the MEDAS [42], and it consists of 28 items divided into three blocks. The first two blocks are dedicated to estimate food consumption frequency and Mediterranean dietary habits, such as the previous MEDAS index [43]. The third block consists of six items that include information about PA (more than 150 min/week or 30 min/day jogging, walking quickly, dancing or doing aerobics) and social habits (time spent sitting, watching television or in front of a computer, sleeping or socializing with friends). The MEDLIFE index is the first to measure other variables beyond food consumption that are part of the Mediterranean lifestyle and it is expected to help in the refinement of association testing between metabolic diseases and diet/lifestyle, as well as in the improvement of measuring adherence to a Mediterranean lifestyle. 

Some authors have proposed that more complex and sophisticated statistical methods may help monitoring the adherence of patients to a nutritional intervention, which would lead to a more accurate detection of the potential associations between dietary interventions and metabolic improvements. In this regard, Sevilla-Villanueva et al. [49] have recently reported that adherence evaluation through trajectory analysis allows researchers to observe how study participants evolve during a nutritional intervention depending upon their assigned nutritional group. This artificial intelligence-based approach considers an initial classification of individuals according to the Integrative Multiview Clustering [50], which uses 65 parameters divided in two blocks to group individuals. These two blocks are the baseline block, describing the health condition (biometric measures, tobacco and drug consumption, socio-demographic characteristics, diseases and biomarkers) and the habits block, which describes food habits and PA. This clustering process is performed at the beginning and at the end of the study, creating a trajectory map showing how the individuals belong to one or other final class by observing changes in diet indicators and depending on the initial state and the assigned intervention. Adherence to the intervention was tested in a randomized, parallel, controlled clinical trial with three dietary interventions (Mediterranean diet plus virgin or washed olive oil, and a control group with habitual diet) [51] where the previously mentioned MEDAS was used to assess individual diet scores [42]. By using this approach, researchers are able to unmask dietary changes within a given intervention group and to discriminate participants according to their particular diet trajectories during the study, and not only by their assigned intervention groups. This type of study allows a more specific evaluation of adherence and a more accurate characterization of the impact of the intervention. In any case, the application of these algorithms will likely continue to be influenced by inadequate self-reported-based estimates of energy intake that are, after all, the input parameters of such sophisticated algorithms [52]. In this sense, a recent study has revealed that energy intake under-reporting keeps being a major concern in nutrition research, regardless of self-reporting method [53]. In this study, a total of 200 men and women from the SCAPIS study (Swedish CArdioPulmonary bioImage Study) [54], and aged 50–64 years were recruited and invited to complete a rapid FFQ (the MiniMeal-Q) and a 4-day web-based food record tool (the Riksmaten method). Reported energy intake by the MiniMeal-Q and the Riksmaten method were tested against total energy expenditure measured with the double-labelled water technique in 40 participants. Both methods are widely used in national dietary surveys in Sweden and in large-scale epidemiological studies, and have been partially validated. Results of this study showed that both methods displayed a similar degree of energy intake under-reporting, with a reporting accuracy of 80% and 82% for the for MiniMeal-Q and the Risksmaten methods, respectively [53]. 

Aiming at a better standardization of adherence monitoring in restricted and free-living individuals, self-reported assessment methods should then be used with caution, and priority should be given to the development of alternative techniques to assess food and energy intake. In this regard, new methods to measure food consumption in a more accurate way are emerging and being validated. A food image-based method, called the Remote Food Photography Method (RFPM) [55], has been recently validated for measuring energy and nutrient intake [56] and has been proposed as a cheap, easy, and reliable method for detecting individual adherence better than classical FFQ. This method involves participants capturing images of their meals and plates waste with a phone camera. These images are further sent to a server where energy and nutrient intake are estimated by validated methods [55]. Another method based on wrist motion tracking aims to give consistent energy intake measurements from daily living by monitoring food bites thanks to a wearable (watch-like) device coupled with a micro-electro-mechanical gyroscope [57]. Although these methods still need to be fine-tuned, they appear to be promising for the optimization of dietary monitoring focused on estimated energy intake and to assess adherence to a nutritional intervention. 

Innovative and sophisticated tools to estimate food and energy intake are expected to be further improved and validated, while more precise devices and techniques such as the above mentioned must be developed. Further research is also needed to test whether these *high-tech* methods can be widely used in free-living subjects [58].

## 3. Food Behavior

### Foodstyle Monitoring

In addition to the measurement of total food intake, additional key aspects concerning precision nutrition that must be considered are, for instance, the frequency at which we consume foods throughout the day, the time we have lunch or dinner, and our snacking habits. Again, relying on methods able to collect accurate and valid clinical observations are key priorities as we strive to obtain reliable research results that will ultimately lead to unbiased interpretations. 

Innovative technologies in this area are being developed, such as the Universal Eating Monitor (UEM), a table-embedded scale able to precisely quantify the amount of food consumed by a given person over time [59]. Initially conceived to monitor unrestricted eating, currently existing algorithms can be used only under restricted laboratory conditions. Nevertheless, the ability of the UEM to monitor different eating behavior parameters such as eating rate, bite size or food-to-drink ratio makes this tool a potentially useful device in precision nutrition. Accordingly, the Automatic Ingestion Monitor (AIM) is a wearable device designed to monitor the food intake behavior, such as snacking, night eating or weekend overeating, and analyze eating behavior in free living conditions [60]. In this regard, the AIM uses three different sensors (jaw motion, hand gesture and accelerometer) that allow obtaining reliable eating behavior measurements. These systems are two examples of how technology can be implemented to account for inter-individual differences in feeding behavior. 

One important aspect of food behavior lies in its interaction with the circadian system, a physiological internal clock working autonomously with rhythms and oscillators synchronized by external time cues, and regulating a variety of physiological functions [61]. Several authors have already shown the relevance of the circadian system in human nutrition. Results from the ONTIME study, a clinical trial focused on the interaction between meal timing, genetics and weight loss showed that carriers of variants at the *PLIN1* locus exhibited lower weight loss within individuals assigned to the group of late lunch eaters (after 15:00), as compared to early lunch eaters (before 15:00) (7.21 ± 0.67 kg vs. 10.63 ± 0.56 kg; *p* = 0.001) [62]. Other food behaviors, such as frequent snacking have also been pinned down to genetics. Garaulet et al. reported that carriers of *PER2* variants displayed extreme snacking, suffered from diet-induced stress and bored-eating, among other behavior atypical patterns [63]. Results of two other recent studies have underscored the relevance of genes linked to the circadian clock in scheduled food behavior. For instance, significant interactions between specific gene variants within the *CLOCK* [64] and the *CRY1* [65] circadian genes, with low-fat diet and carbohydrate intake, respectively, have been identified.

In the first study, the interaction between SNPs at the *CLOCK* locus (rs1801260, rs3749474, rs4580704) with a Mediterranean diet and a low-fat diet was tested in 897 patients with coronary heart disease from the Coronary Diet Intervention with Olive Oil and Cardiovascular Prevention (CORDIOPREV) clinical trial (ClinicalTrials.gov: NCT00924937). After 12 months of intervention, a significant interaction was found between rs4580704 and low-fat dietary pattern for high sensitivity C-reactive protein (hsCRP) levels and the ratio high-density lipoprotein cholesterol/apolipoprotein A1 (HDL/ApoA1). Specifically, after the low-fat diet intervention, rs4580704 major allele carriers (CC) displayed a significant decrease of CRP levels, as compared to minor allele carriers (GG + CG) (~42% vs. ~12.5%; *p* < 0.001) and increased HDL/ApoA1 ratio (~4% vs. ~1.2%; *p* < 0.029) , whereas no changes were observed between genotypes after the Mediterranean diet intervention, thereby suggesting that some metabolic disturbances, such as inflammation or dyslipemia, may be improved with personalized nutritional advices based on the genetic background of circadian rhythm [64].

On the other hand, a SNP (rs2287161) at the *CRY1* locus was tested for interaction with carbohydrate intake in predicting insulin resistance [65]. Results showed that increased carbohydrate intake led to a significant increase of fasting insulin (β_interaction_ (SE_interaction_) = 0.0040 (0.0015); P_interaction_ = 0.007) and the homeostatic model assessment of insulin resistance (HOMA-IR) (β_interaction_ (SE_interaction_) = 0.0040 (0.0016); P_interaction_ = 0.011) only among individuals homozygous for the rs2287161 rare allele. The initial results found in the Mediterranean population of 728 subjects following a Mediterranean diet were further replicated in a North American population of 820 subjects participating in the Genetics of Lipid Lowering Drugs and Diet Network (GOLDN) study. 

These and other findings reviewed by Asher & Sassone-Corsi and Oike et al. [66,67] highlight the relevance of chrono-nutrition, i.e., the study of how food components interact with circadian clocks and how meal times affect metabolic processes [67], in the application of precision nutrition. Concretely, these findings point to circadian genetic variability as a relevant factor to be considered when developing scheduled and personalized nutrition programs aimed to face metabolic disorders associated with obesity.

## 4. Precision Physical Activity 

### Physical Activity: A Key Factor to Proper Precision Nutrition

There is a wide consensus in the literature that a sedentary lifestyle is one of the main factors contributing to the epidemic of cardiometabolic diseases [68]. Monitoring of PA should be then considered as a central factor when approaching precision nutrition. In words of Betts and González: *An optimal diet can therefore be personalized not only to what an individual is currently doing but to what they should be doing* [1]. In this context, Bouchard et al. have shown that besides the inter-individual variability in the beneficial response to a PA intervention regarding cardiovascular disease (CVD) and type 2 diabetes (T2D) risk factors, some individuals may even experience negative responses, such as a decrease in plasma HDL-C or an increase in systolic blood pressure, fasting plasma insulin and plasma triglyceride (TG) levels [69]. Thus, tailored dietary recommendations should take into account the PA profile of individuals, which will open the door to more integrative interventions, including personalized PA prescriptions. Moreover, not only the inter-individual variability in PA rates is relevant when tailoring nutritional advices, but even greater is the within-individual PA variability with time. In this regard, a recent study has shown the relevance of accounting for day-to-day individual variability of insulin and glucose levels in response to a standardized PA intervention. In this study, 171 sedentary, middle-aged abdominally obese adults were randomly assigned to four exercise groups (non-exercise, a low-amount/low-intensity, high-amount/low-intensity and high-amount/ high-intensity). The intervention consisted of walking on a treadmill five times per week at the required intensity (relative to the cardiorespiratory fitness) for 24 weeks. The day-to-day variability was calculated as the square root of the sum of squared differences of repeat measures (glucose and insulin baseline and 24-week levels in the control group), divided by the total number of paired samples and multiplied by two. Taking into account this individual variability, approximately 80% of the participants did not improve glucose and insulin levels, independently of the PA intensity, underscoring the need for a more comprehensive assessment of the PA-derived metabolic outcomes [70]. This study stressed that within-subject variation must be accurately assessed when evaluating the inter-individual differences in precision nutrition approaches. In this regard, Atkinson & Betherman [71] have proposed a logical framework to identify true inter-individual differences after an intervention, as well as to evaluate their clinical relevance. Such approach includes a comparator arm where standard deviation from the intervention arm should be compared to for the identification of reliable differences among participants.

Recent approaches have started to scrutinize the potential role of PA in previously detected genetic associations with obesity and related metabolic disturbances. For example, it has been reported that a sedentary behavior, estimated as prolonged television watching, accentuates genetic predisposition (measured as GRS) to increased BMI in two prospective cohorts, the Nurses’ Health Study and the Health Professionals Follow-up Study [72]. Specifically, an increment of ten points in the GRS was associated with 0.8–3.4 kg/m^2^ higher BMI across the different categories of television watching (1–40 h/week; P_interaction_ = 0.001). Recent findings have also reported that the impact of gene variants within *FTO* gene, the first and most strongly obesity-associated gene [73,74], on obesity development is in fact attenuated by PA, i.e., the increase in BMI is 76% more pronounced in inactive individuals carrying the risk allele (P_interaction_ = 0.004) [75]. Additional studies have also reported a protective effect of PA (assessed using self-administered questionnaires) on the impact of obesity-associated genetic variants in the form of aggregated GRS [76,77]. Results from Li et al. [76] revealed that the genetic predisposition to obesity in individuals with high-risk GRS could mitigated by higher levels of PA, as illustrated by BMI differences in physically active vs. sedentary participants. More specifically, the BMI difference between high- and low-risk GRS individuals in the sedentary group amounted to 0.74 kg/m^2^, whereas this difference was 0.41 kg/m^2^ in the physically active group. These results were also replicated in a meta-analysis of 11 cohorts [77], where a significant but weak association was reported (0.65 kg/m^2^ vs. 0.53 kg/m^2^). Despite the adequate power to detect small effects and the large number of participants in above-mentioned studies, over 20,000 and 100,000 individuals, respectively, gene × PA interactions are not strong enough to establish causal relationships between increased PA and decreased risk of genetic predisposition to develop obesity, or to use them in clinical practice, as reported by Ahmad et al. [77]. Likewise, a more recent GWAS meta-analysis of 200,452 subjects from 60 previous studies analyzing gene-PA interactions revealed 11 novel loci associated with adiposity, suggesting that accounting for PA could facilitate the uncovering of novel biological determinants of obesity [78]. Nevertheless, the search for PA interactions with obesity-associated loci only provided significant results with the *FTO* gene, showing a decrease of 30% of *FTO* effect in active as compared to sedentary subjects [78]. Although it has been hypothesized that highly penetrant genetic variants may be less influenced by environmental factors [79], it is important to point out that PA is most often estimated by self-reported questionnaires in population studies. In this regard, it is worth highlighting that the majority of studies included in this meta-analysis used self-reported PA data (self-administered or interviewer-administered questionnaires) instead of objective measures (only two studies measured PA by accelerometry), and PA was finally treated as a dichotomous variable (active and inactive individuals) to harmonize this parameter, with the resultant loss of power to detect associations.

It then becomes crucial to replicate these findings with direct and objective measures of PA. In this sense, the recent use of accelerometers to objectively measure PA levels has consistently revealed that both BMI-associated GRS [80] and *FTO* impact on obesity susceptibility [81] are attenuated by higher levels of objectively measured PA. With direct PA measurements, results from the *FTO*-related work [81] are similar to previous findings [75,82], but the attenuation of *FTO* impact on obesity-associated features, such as BMI and WC, is quantitatively more important. These findings could be explained, according to the authors, by the higher precision of PA measurements and its ability to accurately categorize PA intensity into light, moderate or vigorous.

**Figure 3:**
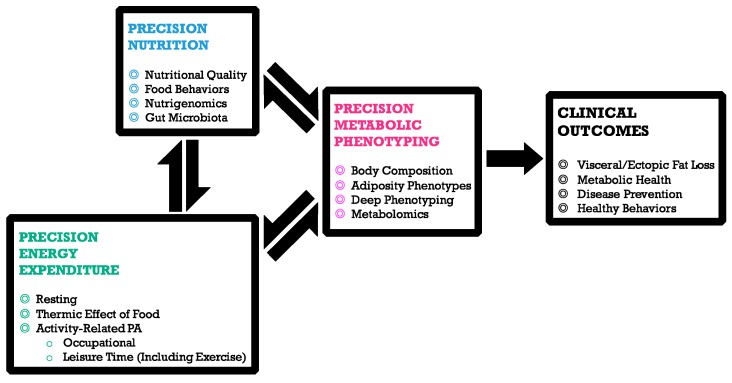
Precision nutrition features and their relationships. PA: physical activity.

These findings underscore the importance of a reliable assessment of PA for a more accurate interpretation of its potential modulating effect on the association between diet and health outcomes. Up to now, motion sensors such as accelerometers could be considered as the gold standard to obtain accurate PA measurements [83], and their use in biomedical research in increasing [84,85]. A recent systematic review of the use of accelerometers for measuring PA under free-living conditions revealed that triaxial accelerometers were the most commonly used, followed by biaxial and uniaxial [86]. Among triaxial accelerometers, the most used models in longitudinal assessment of PA in studies related to health and disease [87] were ActiGraph GT3X (ActiGraph LLC, Pensacola, FL, USA) [70,88] and TracmorD (DirectLife, Philips Consumer Lifestyle, Amsterdam, The Netherlands) [81,89]. Nevertheless, these methods have some limitations for large prospective epidemiological studies, e.g., intrusiveness, elevated cost or specialized training for an efficient use [90], limitations that researchers should try to overcome in order to develop precision nutrition approaches that integrate the important notions of energy expenditure and energy balance. In this regard, together with an accurate knowledge of dietary habits, food behaviors, genetics and gut microbiota factors, as well as a precise metabolic phenotyping, precision energy expenditure measurements, including resting energy expenditure (REE), thermic effect of food and activity-related PA should be considered when implementing precision nutrition approaches, as depicted in Figure 3. Regarding activity-related PA, a multidimensional representation of PA (including factors such as occupational PA, sedentary time and leisure activities) has been recently proposed as a way to provide a more comprehensive picture of PA, reducing the bias associated to a unidimensional approach solely based on PA *per se* [91].

## 5. Deep Phenotyping

### High-Quality Phenotypes to Stratify Obesity

The need for precise measures to refine phenotypes emerges as a key pillar to understand inter-individual variability observed for certain pathologies, as well as over time variability for an individual [92]. This is of special relevance when the impact of a given diet or lifestyle advice on specific phenotype features is the pursued goal. Accurate and well-defined disease stratification, taking into account phenotypic heterogeneity, is then required in order to obtain reliable associations in nutritional interventions (Figure 3). 

Complex diseases mean complex phenotypes. This is the case of most metabolic disorders, such as obesity, CVD or T2D. The development of new tools or methods with the ability to stratify and distinguish different phenotypes in terms of etiology, severity or underlying mechanisms thus represents a challenge in the field of precision nutrition. In this regard, although obesity is a condition associated with increased risk of T2D, CVD and other metabolic complications, a significant proportion of individuals with excess body weight are characterized by a much healthier metabolic risk profile than what could be expected form their excess adiposity [93], thereby suggesting that BMI does not reflect the actual health status of an individual. On the other hand, individual with a normal BMI may still be characterized by metabolic dysfunction if they carry excess visceral or hepatic fat. Since excess visceral adipose tissue (VAT) accumulation and adipose tissue dysfunction are tightly related to the development of obesity-related metabolic complications [94], it has been proposed that the combined measure of WC and plasma TG levels may represent an inexpensive and useful biomarker of both VAT accumulation and dysfunction, as well as a potential predictor of T2D and CVD risk [95,96]. Results from a study including 21,787 apparently healthy individuals, followed for approximately 10 years as part of the EPIC-Norfolk prospective population study, have shown that the hypertriglyceridemic-waist phenotype (the combination of elevated WC and plasma TG levels) was associated with increased risk (unadjusted hazard ratio—UHR) for coronary artery disease in both men (*UHR* = 2.40 (2.02–2.87)) and women (*UHR* = 3.84 (3.20–4.62)) [97].

Several imaging studies have now shown that VAT accumulation has a more deleterious effect than subcutaneous adipose tissue (SAT) on metabolic health [94,98]. Assessment of VAT accumulation represents a challenge when stratifying subjects with abdominal obesity. In this regard, a recent study has shown that epigenetic factors, such as DNA methylation marks, could discriminate VAT from SAT after weight loss surgery [99]. Given that VAT biopsies represents an invasive technique, there is a need to find surrogate biomarkers in more accessible tissues such as blood, allowing a better characterization of obesity in addition to the traditional clinical outcomes, such as BMI or WC. Recent studies have also suggested that whole genome differential methylation patterns derived from blood leukocytes (BL) may be used as surrogates of those derived from VAT. More specifically, a set of differentially methylated cytosine-phosphate-guanine (CpG) sites, common in VAT and BL, were shown to successfully discriminate men with or without metabolic syndrome [100]. These and other results suggest that BL methylation levels could be a good marker of VAT DNA methylation [101], and could then be used to determine the effect of a nutritional intervention on the epigenetic profile, and therefore on metabolic health related to VAT accumulation. Thus, knowledge of epigenetic variations predictive of metabolic complications among individuals with obesity could be of considerable relevance to the field of precision nutrition. For instance, recent studies focusing on DNA methylation differences between responders and non-responders to a weight loss intervention (energy restriction or bariatric surgery) [102,103,104,105] suggest that these epigenetic marks may be used as biomarkers to identify high-risk individuals who may be targeted in personalized nutritional programs focused on prevention, management and treatment of obesity.

Robinson has defined deep phenotyping as the precise and comprehensive analysis of phenotypic abnormalities in which the individual components of the phenotype are observed and described [106]. Traditional risk factors for T2D and CVD, such as blood pressure, lipid profile or BMI, are not always representative enough of a given health condition. Rather, in some instances, a thorough, individualized and precise evaluation of a number of metabolic parameters, e.g., continuous glucose monitoring in T2D, could be required [106,107]. This means that a much more detailed phenotyping is required in order to capture the diverse, interindividual and time-dependent, variability of disease manifestations for a better disease stratification.

An example of this extensive phenotyping is the Maastricht Study, where a cohort of 10,000 individuals with an overrepresentation of patients with T2D are being surveyed in a regular basis for T2D traditional risk factors, etiology and associated metabolic disturbances [108]. In this study, traditional (hypertension, dyslipidemia, obesity or inflammation status) and advanced phenotyping techniques (body composition by dual energy X-ray absorptiometry, electrophysiology of the heart, ocular pressure, corneal confocal microscopy or lung function evaluation by spirometry) are being used to elucidate the underlying pathophysiology of T2D and associated metabolic disturbances. This large epidemiological study will likely provide important clues on how detailed phenotyping can be extracted and eventually applied in precision nutrition. In-depth phenotyping methods utilized in this study can be divided into four different approaches: exhaustive biobanking for an efficient risk stratification (whole blood for DNA and RNA extraction, 24 h and morning urine, fasting and post-oral glucose tolerance test serum samples), advanced cardiovascular imaging for better knowledge of T2D-associated CVD risk (microvascular assessment by nail fold microscopy, vascular and cardiac ultrasound), use of accelerometry to objectively measure PA and sedentary time, and the study of psychosocial factors through personality questionnaires. The use of advanced and objective measurements (abdominal fat ultrasound, triaxial accelerometry), combined with traditional and self-reported data (FFQ, PA) represents a strength of this study [108] and a step forward in precision nutrition. 

As previously outlined in the *Precision nutrition* section, a similar strategy was carried out by Zeevi et al. in their work about prediction of glycemic responses and personalized nutrition [33]. This work represents a proof-of-concept for the feasibility of individualized prediction of the response to a meal by combining traditional and in-depth measurements, such as FFQ, food diaries, blood tests and microbiome profiling, being able to assign specific diets allowing to successfully lower post-meal blood glucose.

The strengths of deep phenotyping span from providing a much more detailed picture of a given pathology, which allows a better clinical decision-making, to deepening the knowledge of mechanisms governing the progression of the pathology, which improves the evaluation of intervention outcomes. Thus, despite its limitations, such as the high cost, the need for intensive clinical measurements, or the necessity of accurate evaluation by trained professionals, deep phenotyping represents an essential tool for the optimal stratification of diseases to easily and efficiently manage each subtype according to its particular characteristics [107,109]. Translational and precision nutrition approaches shall then profit from this specific and *fine-grained* phenotype information to adequately apply novel and personalized dietary advices.

## 6. Metabolomics

### Towards a Better Characterization of Eating

The accurate understanding of how nutrients are metabolized, and how these metabolites are able to illustrate the body’s response to a diet has been previously addressed in many studies [110]. Regarding precision nutrition, metabolomics stands as a cornerstone in the knowledge of the real impact of foods on an individual’s health. By identifying food-derived biomarkers, scientists can now determine how different individuals metabolize the same foods distinctly, and how such food products or metabolites may further influence health outcomes in different healthy or unhealthy situations, as well as in atypical conditions, such as intolerances or allergies. In this regard, the standardization of reference values for metabolites is necessary for the further use of them as food-derived biomarkers in the setting of precision nutrition. A recent study carried out in 800 French healthy volunteers where 185 plasma metabolites were analyzed has established a reference dataset for the majority of them [111]. Moreover, this study allowed to differentiate *normal* metabolomes between men and women, elderly and young subjects, and to determine the main sources of variation between population subgroups. As an example, results of this study revealed that individuals with high total cholesterol levels were also characterized by higher plasma sphingomyelins and phosphatidylcholine concentrations [111].

As previously mentioned in the *Dietary habits* section, objective measurement of the adherence to a dietary pattern remains a major challenge of precision nutrition. Recent advances in metabolomics offer a glimpse into promising avenues for better eating characterization. For instance, it could be possible to identify individual foods or nutrients, such as polyphenols, wheat, sugar-sweetened beverages or walnut consumption [112,113,114,115]. A further step in metabolomics usefulness is to test its ability to determine the overall picture of an individual food consumption [116]. In this sense, spectroscopic profiling of urine with the use of proton nuclear magnetic resonance (^1^H-NMR) has recently been validated for the objective measurement of an overall dietary pattern [117]. The 19 participants of this randomized, controlled, crossover trial were assigned to four dietary interventions with a stepwise variance in concordance with the World Health Organization (WHO) healthy eating guidelines. Adherence to the intervention was strictly monitored by food and plate waste weighing, and urine samples were collected daily over three time periods. A global metabolomic profiling with a combination of 16,000 spectral variables was used to generate representative metabolite patterns relevant to each diet. Systematic differences were found in metabolomic profiles between diets 1 and 4 (the most and the least concordant diets to the WHO guidelines). Among individual metabolites, significantly higher concentrations of hippurate (fruit and vegetables), tartrate (grapes) or dimethylamine (fish) were found in the urine of participants assigned to diet 1, as compared to participants assigned to diet 4, while others were significantly lower, such as carnitine (red meat). The ability of this technique to discriminate metabolomic profiles of participants and classify them according to their assigned healthy or unhealthy diet during a nutritional intervention was further successfully validated in free-living populations [117]. However, the low specificity and sensitivity of this method in the discrimination of dietary patterns must be addressed, and some other challenges such as its potential to capture diet dynamics thorough long-term studies have to be considered [118]. The same ^1^H-NMR technique has also been used to characterize the metabolomic profiles of a whole meal [119]. In this study, a cereal breakfast and an egg and ham breakfast were distinguished by identifying acute metabolomic fingerprints and key discriminatory metabolites in postprandial urine samples. Concretely, phosphocreatine/creatine, citrate and lysine were at higher concentrations after an egg and ham breakfast, whereas erythrose showed a higher concentration after a cereal breakfast [119].

Metabolomics has also been successfully applied in the development of novel population classification methods according to the metabotype, which stands for a group of individuals with similar metabolic profiles, and therefore represents a pillar of *deep phenotyping* [120]. One of the advantages of stratifying population according to the metabolic profile (metabotyping) is the possibility to scale precision nutrition advices to relatively uniform groups of individuals. As an example, since low-grade inflammation is known to be an important factor in insulin resistance development, the search for nutritional strategies focused on alleviating the inflammatory state becomes an attractive approach for precision nutrition [121,122,123]. In this regard, recent studies based on baseline metabolic profiles, e.g., plasma lipoprotein and fatty acid profiles, cardiometabolic biomarkers or insulin and glucose fasting and postprandial levels, have revealed the ability to *a priori* discriminate between responders and non-responders to a specific treatment or nutritional intervention, as recently reviewed by Riedl et al. [124]. Moreover, preliminary results from a dietary intervention in overweight and obese adolescents (ClinicalTrials.gov: NCT01665742) suggest that the beneficial effects of anti-inflammatory supplements (omega-3 polyunsaturated fatty acids—*n*-3 PUFA—vitamin C, vitamin E, and polyphenols) on insulin sensitivity are limited to the patients with the least favorable metabotype, whose different components (high HOMA-IR and cholesterol levels) also serve as independent predictors of nutritional supplementation outcomes [125].

Emerging evidence suggests that both pre- and post-metabolic profiles in patients undergoing a nutritional intervention can provide valuable information about capabilities of metabotypes to predict a given response to nutrients, and to determine the influence of individual foods, whole meals and dietary patterns on plasma metabolite levels. Thus, the potential of metabolomics has to be further explored in precision nutrition approaches.

## 7. Microbiota Phenotyping

### Diet-Gut Microbiome Interplay 

Gut microbiota profiling is becoming a top priority in nutritional interventions, and the impact of specific dietary factors on the ecological diversity of the gut is currently the subject of many ongoing investigations. The development of nutritional interventions based on individual profiles are focused on optimizing gut microbial composition, both richness and diversity, and emerging evidence suggests that gut microbiota profiling should be included as a key feature of precision nutrition [126]. In fact, both composition and diversity of gut microbiota have been identified as potential risk factors for the development of several metabolic disorders including the metabolic syndrome, T2D and CVD [127]. In this regard, the previously discussed study of Zeevi et al. [33] is an example of how gut microbiome profiling could represent a tool allowing accurate glucose response prediction after a meal. In this study, gut microbiota profiling was performed in stool samples of the entire cohort of 800 participants by 16S rRNA and metagenomics sequencing. Numerous microbiome features of composition and function were then integrated into the postprandial glucose response prediction algorithm. The analysis of the contribution of each factor to algorithm predictions revealed 21 beneficial and 28 non-beneficial (decreased or increased predicted postprandial glucose response, respectively) microbiome-based features. For instance, *Eubacterium rectale* abundance was mostly beneficial, whereas *Parabacteroides distasonis* was found to be non-beneficial by the prediction algorithm. Other studies have also highlighted the potential relevance of gut microbiota in tailoring diets. For instance, the FRUVEDomics Study, a behavioral interventional trial (ClinicalTrials.gov ID: NCT03115866), aims at identifying metabolomic and microbiome risk factors that may be subjected to modification through a nutritional intervention, mainly based on increasing fruit and vegetable consumption in young adults at risk for the metabolic syndrome. For that purpose, 36 participants were randomized into three intervention groups. The first one was based on a dietary intake of 50% fruit and vegetables, and the other two groups were based on the same 50% fruit and vegetables plus low refined carbohydrate or low fat. Although it is expected that this trial will be completed in 2019, preliminary results have suggested that individuals with a higher risk of developing metabolic syndrome also exhibited a higher *Firmicutes* to *Bacteroidetes* ratio before the intervention [128,129,130]. Other than being able to identify different combinations of diets to improve metabolic health, this type of trial is an example of group-based nutritional interventions (at-risk metabolic syndrome young adults). Studies like this have the potential of revealing novel biomarkers, both metabolomic and issued from microbiome profiling, allowing a phenotype refinement that could eventually be used in further individually tailored studies.

A relevant aspect of the gut microbiome is the fact that its composition and diversity can be modulated by host genetic makeup [127]. But even more relevant for the precision nutrition field is the fact that the interaction between diet and host genetic background is also able to modulate the composition of the gut microbiota. A recent study aiming at documenting the impact of host genetics on the gut microbiome found that, in addition to 9 novel loci associated with gut microbial taxonomies and other chromosomal regions related to food preferences, gene-diet interactions regulate *Bifidobacterium* abundance [131]. This study was carried out in three independent Dutch population cohorts: a discovery cohort of 1539 individuals, and two replication cohorts of 534 and 105 individuals, respectively. Interestingly, a functional variant at the lactase (*LCT*) locus, tightly associated with lactase persistence in Europeans [132], was associated with higher abundance of *Bifidobacterium*. Dairy product consumption was not altered significantly by this haplotype, nor by increased *Bifidobacterium* abundance. Nevertheless, the interaction between this haplotype and the intake of dairy products was associated to *Bifidobacterium* abundance [131]. These results pointed out the potential modulation of the microbiome through the interaction between diet and genetic makeup as a target to be considered in further precision nutrition studies [133].

Other examples highlighting the relevance of gut microbiota in precision nutrition have reported its role in the relationship between red meat consumption and the development of atherosclerosis and CVD [134,135]. In these studies, increased fasting plasma levels of trimethylamine (TMA), produced by gut microbiota metabolism, and its proatherogenic metabolite trimethylamine-*N*-oxide (TMAO) were observed in mice and humans, concomitant with increased risk of atherosclerosis, after oral intake of l-carnitine [134] and phosphatidylcholine [135], both having red meats as a major source. Interestingly, as suggested by Zmora et al. [136], these pieces of work suggest that the general recommendation of reducing the intake of red meat [137] may be better focused on subjects with gut microbial configurations more prone to metabolize such nutrients into proatherogenic species. This study also revealed that the association between red meat intake and mortality could be due to a certain extent to gut microbiota-derived metabolites. Other general recommendations, such as the substitution of sugar consumption by artificial sweeteners, have also revealed that such an approach may not potentially be beneficial for a population subgroup, as reported by Suez et al. [138]. In this study, an increase in the intake of sweeteners led to the development of glucose intolerance in the subgroup of individuals having a sensitive gut microbiota [138]. However, given the high dose of sweetener used (5 mg saccharin/kg body weight—FDA’s maximum acceptable daily intake) and the limited number of participants (*n* = 7), the results issued form the study of Suez et al. [138] are still controversial and have generated a broad discussion in the field [139,140]. Although there is mounting evidence supporting the impact of sweeteners on microbiota in rodents [141], larger studies in humans are still needed. In this regard, a recent study has shown that gut bacterial diversity could be affected by recent (four-day food record) sweetener consumption (aspartame and acesulfame potassium) [142]. 

In summary, recent findings summarized herein suggest a link between microbiome biomarkers and nutritional intervention outcomes [33], the ability of gene-diet interactions to modify gut microbiota composition [131], and the existing link between food consumption, disease development and gut bacteria diversity [134,135]. Altogether, these findings suggest that gut microbiota should be considered when designing individualized nutrition advices. 

## 8. Recent Advances in Precision Nutrition

### 8.1. From Nutrigenomics to Tailored Nutrition

Genetics have been frequently considered in association studies as an independent factor predisposing to obesity, increased adiposity, T2D and CVD [143,144,145,146]. Both GWAS and candidate gene studies have mostly focused on the impact of genetics on metabolic health [147,148,149,150]. Although this strategy has identified strong statistical associations, knowledge about the underlying molecular mechanisms affected by these genetic variants, and allowing to interpret their clinical relevance in the development of such pathologies is still scarce [151]. As already mentioned in this review, the physiological consequences of genetic variants and of their interactions with nutrient intake and other lifestyle factors, such as PA [76,77,78] or dietary habits [19,20,28] have been carried out in the field of precision nutrition.

Besides the fact that interesting findings have emerged from these studies, more ambitious, comprehensive and overarching strategies are currently broadening the knowledge about factors involved in the different response to a given nutritional intervention. This is the case of two large randomized control trials, PREDIMED and Food4me. 

### 8.2. PREDIMED

Given that previous studies have reported that increasing adherence to the Mediterranean diet has beneficial effects on cardiovascular health [152,153], the PREDIMED study was designed as a multicenter, randomized, controlled trial to determine the impact of this diet on cardiovascular outcomes in participants at high cardiovascular risk [46]. The general guidelines to follow the Mediterranean diet were provided to participants as a personalized dietary advice according to their prior adherence to this type of diet, evaluated by the previously mentioned 14-item MEDAS questionnaire [42]. These guidelines consisted in abundant use of olive oil for cooking, generous consumption of vegetables and fresh fruits, legumes, fish or seafood, nuts and seeds, selection of white meats instead of red and processed meats, and cook regularly with tomato, garlic and onion. Further recommendations were focused on reducing the consumption of certain foods, such as butter, sugar-sweetened beverages, pastries or French fries. The unique aspect of this study was the intensive utilization of a constellation of omics techniques (transcriptomics, genomics, epigenomics or metabolomics), the analysis of intermediate phenotypes (plasma lipid concentrations, inflammation markers or blood pressure) and end points (myocardial infarction, stroke, and death from CVD causes), and a robust dietary adherence assessment with a validated 14-item questionnaire [42,154], all of them key pillars in in the field of precision nutrition [155]. The plethora of biomarkers analyzed in the PREDIMED study ranged from genetic, epigenetic and transcriptomic, to proteomic, lipidomic and metabolomic determinants, allowing an in-depth evaluation of the effect of Mediterranean diet on the basis of an integrated framework. For instance, the use of this integrative approach allowed further analysis focused on the genetic makeup, such as the TG-lowering effect of a genetic variant (rs3812316) at the *MLXIPL* locus. Such TG-lowering effect was strengthened in subjects having a high adherence to the Mediterranean diet (*OR* = 0.63 (0.51–0.77); *p* = 8.6 × 10^−6^), as compared to those having a low adherence (*OR* = 0.88 (0.70–1.09); *p* = 0.219), which also enhanced the protective effect against myocardial infarction among carriers vs. non-carriers of this SNP (*HR* = 0.34 (0.12–0.93); *p* = 0.036) vs. (*HR* = 0.90 (0.35–2.33; *p* = 0.830), respectively). [156]. Previous nutritional interventions focused on gene-diet interactions have also revealed that TG levels can be modulated by diet depending upon the genetic background [157]. In that study, 210 participants received a daily supplementation of *n*-3 PUFA (5 g of fish oil) during 6 weeks to investigate the interindividual variability in plasma TG response to such supplementation. The GRS built with 10 SNPs showing significant frequency differences between extreme responders (the most significant reduction in plasma TG levels) and non-responders (no change in plasma TG levels) explained 21.5% of the variation in TG response. Another example of the distinctive effect of the Mediterranean diet depending on genetic background was found in a recent case-control study with more than 7000 participants, with or without T2D, issued from the PREDIMED study [158]. A significant interaction was observed between the adherence score to the MEDAS 14-item questionnaire and a GRS formed by two SNPs at the *FTO* and *MC4R* loci in determining T2D risk (P_interaction_ = 0.006). Specifically, carriers of the rare alleles of these two loci had higher T2D risk when adherence to the Mediterranean diet was low, but this association disappeared as adherence increased [158].

Metabolomic tools such as liquid chromatography and mass spectrometry are also being used in the PREDIMED study to characterize walnut or cocoa consumption under free-living conditions [115,159], providing a better assessment of dietary exposure to specific nutrients, which might be successfully applied in precision nutrition for the assessment of complex dietary patterns. More examples of gene-diet interactions, metabotyping and the Mediterranean diet impact on gene expression, epigenetic or lipidomic biomarkers emerged from the PREDIMED study are extensively reviewed by Fitó et al. [155].

### 8.3. Food4Me

Advanced tools and innovative approaches in nutrition assessment are being developed within the Food4Me project (food4me.org). This project, which started in 2011, is carried out by an international consortium focused on the translation of current nutrition knowledge into tailored diets and nutritional advices by meeting three fundamental elements: reliable dietary intake assessment, deep phenotyping (metabotyping), and universal genotyping [160]. The Food4Me project represents a step forward in the potential application of precision nutrition, since in collaboration with stakeholders from different areas (consumers, industry, regulators, etc.) it also seeks to report the attitudes and beliefs of study participants [161], as well as the legal and ethical aspects of this type of nutritional interventions.

Regarding dietary assessment, an innovative web-based tool was tested in a large randomized, controlled trial where participants were randomly assigned to intervention groups for a 6-month period [162]. Nutritional interventions were divided into conventional dietary advice, personalized nutritional advice based on baseline diet and phenotype and, finally, a third group where personalized nutritional advice where based on diet, phenotype and genotype. Baseline diet was evaluated by means of the validated Food4Me online-FFQ [163,164] and phenotypes were assessed by using self-reported anthropometric measurements (body weight, height and upper thigh, waist and hip circumferences) and metabolic parameters (glucose, total cholesterol, carotenoids, *n*-3 fatty acid index and 32 other fatty acids, and vitamin D). Genetic information used for deriving genotype-based personalized nutrition advice was based on loci associated with BMI, weight and WC (*FTO*; rs9939609), *n*-3 PUFA (*FADS1*; rs174546), fat intake (*TCF7L2*; rs7903146), saturated fat (*ApoE*(*e4*); rs429358/rs7412) and folate (*MTHFR*; rs1801133). This type of study design was applied to test the efficacy of personalized nutritional advices for improving consumption of a Mediterranean diet [89], that was estimated on the basis of the PREDIMED 14-item questionnaire [42,154]. In this last study, personalized nutritional interventions were divided as previously mentioned (based on baseline diet, phenotype and genotype), plus on the basis of PA, that was evaluated by using the Baecke questionnaire [165] and accelerometer data. Results from this study revealed that adherence scores to a Mediterranean diet were greater among individuals assigned to personalized intervention groups, as compared to the control group (non-personalized general dietary advice) (5.48 ± 0.07 vs. 5.20 ± 0.05, respectively; *p* = 0.002), with the largest differences found when genotype data was included in the analysis of the intervention group (5.63 ± 0.10 vs. 5.38 ± 0.10, respectively; *p* = 0.029). Similarly, a recent randomized controlled trial illustrated how disclosing genetic information can lead to greater behavioral changes in dietary habits than population-based or general nutritional recommendations [166]. Specifically, sodium intake after a 12-month intervention in participants informed that they possessed a risk allele of the *ACE* gene (associated with increased sodium sensitivity) [17,18], and given a targeted recommendation, was significantly reduced as compared to participants in the control group, who received a general recommendation for sodium intake (mean change in mg: −287.3 ± 114.1 vs. 129.8 ± 118.2, *p*  =  0.008). On the other hand, the intervention group composed of non-risk *ACE* participants and receiving a general recommendation for sodium intake did not show significant differences as compared to the control group (mean change in mg: −244.2 ± 150.2 vs. 129.8 ± 118.2, *p*  =  0.11), suggesting that a targeted nutritional advice based on genotype information impact the intake of specific nutrients in a greater extent than a general recommendation. 

It is worth highlighting that the optimal assessment of nutrient intake remains the foundation for personalized nutritional advice in the Food4Me study [160]. In this regard, dietary data was collected by a validated online FFQ [163,164] and participants received regular feedback with practical advice to improve, increase or decrease, the intake of specific nutrients. Likewise, besides traditional parameters (self-reported BMI and WC), deep phenotyping was performed by means of metabolomic measurements (glucose, cholesterol, carotenoids and lipid profile) collected using a dried blood spot technique [162]. Finally, dietary advice was also controlled regarding individual genotype information, that was referred to five diet-responsive SNPs located within genes linked to different anthropometric (body weight) and metabolic functions (total fat, saturated fatty acids, *n*-3 PUFA and folate) [162]. 

Recent findings from the Food4Me project have revealed promising advances around the three levels stated: dietary intake, deep phenotyping and genotyping. First, it has been reported that a personalized nutritional advice only based on individual baseline diet information (first level) could lead to greater positive changes in nutritional behavior than a conventional dietary advice after a 6-month intervention, i.e., decreased consumption of red meat (8.5%), saturated fat (7.8%) and salt (8.9%), and increased consumption of folate (11.5%), leading to significantly higher Healthy Eating Index (HEI) scores [167,168]. Second, it was also reported that deep phenotyping information could serve as predictor of the response to a nutritional intervention. For instance, baseline fatty acid profiles were able to predict the cholesterol response to a personalized dietary intervention [169]. Finally, regarding genotyping data, although disclosure of information about *FTO* genotype risk had a greater effect on body weight and WC reduction in risk carriers, as compared to the non-personalized control group, these changes were similar to the previously mentioned levels of personalized dietary advice [170]. In line with these results, a previous meta-analysis carried out with data from eight randomized controlled trials revealed that carriage of the *FTO* minor allele was not associated with significant differences in BMI, body weight or WC change in response to a weight loss intervention (dietary, PA or drug-based) [171].

## 9. Conclusions

Altogether, the studies reviewed herein illustrate the most recent approaches to precision nutrition from different perspectives, highlighting the need for an integrative framework that takes into account the richness of innovative tools and methods in this field. This review was an attempt to stress the most important challenges and issues that nutritional science has to overcome in order to successfully translate basic and clinical knowledge into an effective precision nutrition care. 

Up to date, the PREDIMED study and the Food4Me project could be considered as state-of-the-art trials in the field of precision nutrition, and two of the most stimulating wide-scale approaches in this field, that will hopefully provide guidance about how precision nutrition could be used to successfully prevent and manage cardiometabolic disorders. As already mentioned, such integrated approaches have the potential to improve dietary behaviors in an individualized or in a group-based manner, and to generate new and innovative tools, methods and procedures.

It is worth mentioning that although precision nutrition remains in its infancy, it does not take away from the fact that great approaches have been translated into general practice, mainly in the field of nutrigenetics. As extensively reviewed in [172], the large body of evidence supporting this genetic-based approach warrant further progress in this field. At this point, it is important to underscore some limitations encountered by genetic-based nutrition in its translation into general practice, such as the skeptical views of registered dietitians toward genetic testing and the scarcity of such personalized approach in higher education curricula of health professionals [173,174]. In this regard, the feasibility of the whole precision nutrition framework will depend on joint efforts of all actors involved. On one hand, while nutrition professionals are expected to start adopting new diagnosis and follow-up techniques, policy makers should elaborate appropriate policies assuring, among others, an adequate protection of personal information issued from intensive data collection. On the other hand, a substantial part of the task of translating precision nutrition into a widely applicable procedure in nutritional practice remains on private industries, by pursuing the development of precision nutrition tools affordable and accessible to the general population. Thus, as highlighted in the position statement of the International Society of Nutrigenetics/Nutrigenomics [175], ethical and legal aspects around precision nutrition, as well as the built environment and social contexts that influence food consumption have to be considered for a wide and fruitful implementation of this promising concept of *modern nutrition* into the general population.
